# Supplementation of whole grain flaxseeds (*Linum usitatissimum*) along with high cholesterol diet and its effect on hyperlipidemia and initiated atherosclerosis in Wistar albino male rats

**DOI:** 10.14202/vetworld.2018.1433-1439

**Published:** 2018-10-17

**Authors:** H. Srinivasa Naik, Ch. Srilatha, K. Sujatha, B. Sreedevi, T. N. V. K. V. Prasad

**Affiliations:** 1 Department of Veterinary Pathology, College of Veterinary Science, Sri Venkateswara Veterinary University, Tirupati - 517 502, Andhra Pradesh, India; 2 Department of Veterinary Microbiology, College of Veterinary Science, Sri Venkateswara Veterinary University, Tirupati - 517 502, Andhra Pradesh, India; 3 Department of Nanotechnology, Frontier Institute of Technology, RARS, Tirupati, Andhra Pradesh, India

**Keywords:** atherosclerosis, flaxseeds, hepatic steatosis, hyperlipidemia, tissue antioxidants

## Abstract

**Background and Aim:**

Flaxseeds are known to have varying antihypercholesterolemic and antiatherogenic activity due to its lignan secoisolariciresinol diglucoside, alpha-linolenic acid, and omega-3 fatty acids. The beneficial effect of whole grain dietary flaxseed was evaluated experimentally in high cholesterol diet (HCD)-fed Wistar albino rats.

**Materials and Methods:**

Male Wistar albino rats (200 g) were divided into four groups of 12 rats each. Group I rats kept as control and given basal rat chew diet, Group II as positive control for induction of hypercholesterolemia and atherosclerosis by addition of 1% cholesterol and 15% saturated edible oil to the 1000 g of standard rat chew diet (HCD), Group III rats fed with whole grain flaxseed powder at 7.5 g/kg of rat/day in the standard rat chew diet and kept as flaxseed control, and Group IV rats supplemented with flaxseed at 7.5 g/kg of rat/day along with HCD and maintained for 90 days.

**Results:**

Group II rats revealed significantly (p<0.05) higher total cholesterol (TC), triglycerides (TG), low-density lipoprotein cholesterol (LDL-C), and very LDL-C and significantly (p<0.05) reduced levels of high-density lipoprotein cholesterol (HDL-C), whereas tissue antioxidants such as catalase, superoxide dismutase (SOD), glutathione peroxidase (GPx), glutathione reductase (GR), and glutathione S transferase (GST) were significantly (p<0.05) reduced, and lipid peroxidation products of thiobarbituric acid reactive substances (TBARS) level were nonsignificantly (p<0.05) increased in the heart and liver tissues. Flaxseeds supplementation along with HCD significantly ameliorated the serum levels of TC, TG, LDL-C, and HDL-C along with cellular antioxidant enzymes such as catalase, SOD, GPx, GR, GST, and non-significant amelioration of TBARS in the heart and liver tissues compared to Group II rats. Majority of the histopathologically initiated atherosclerotic changes in the aorta and fatty change in the liver of Group II were not observed in the flaxseed supplemented Group IV; however, interestingly proliferation of endothelial cells with new vascular channel formation in the liver and in between cardiac muscle fibers was observed in Group I and Group IV rats.

**Conclusion:**

The present study established the hypercholesterolemia with initiated atherosclerotic lesion in the aorta but unable to establish the atheromatous plaque in the aorta. Flaxseed supplementation along with HCD showed significant antihypercholesterolemic effect and ameliorated the changes of initiated atherosclerosis in the aorta. It needs further studies to explore all the possible beneficial effects and angiogenic properties of flaxseeds in the laboratory animals and human trials.

## Introduction

Hyperlipidemia is accompanied by elevated serum total cholesterol (TC), triglycerides (TGs), low-density lipoprotein cholesterol (LDL-C) and very LDL-C (VLDL-C), and decreased high-density lipoprotein cholesterol (HDL-C) levels [1]. It is associated with cardiovascular diseases (CVD) including coronary heart disease and stroke and is one of the leading causes of mortality in both developed and developing countries, accounting 30% of all worldwide deaths per year [2]. The current reports suggest that by the year 2020, India will have the largest CVD burden in the world [3]. Hyperlipidemia is considered as the primary mediator of a cascade of atherosclerosis [4]. Atherosclerosis is a cardiovascular and fibroproliferative inflammatory disease commonly associated with age and “dietary-related factors” in humans. In animals, atherosclerosis is rarely noticed.

Flaxseeds are the best source of lignans (secoisolariciresinol diglucoside [SDG]), soluble and insoluble dietary fibers, as well as omega-3 fatty acids. Omega-3 fatty acids play their role in reducing the risk of CVDs by reducing oxygen free radicles [5]. Flaxseed SDG has antioxidant, anti-inflammatory, and potent angiogenic and antiapoptotic properties, which plays a role in antiatherosclerosis. The flaxseed fiber is also considered to reduce the blood glucose and cholesterol levels by delaying and reducing their absorption from the intestine [6].

Hence, the present study has been carried out to evaluate the hypercholesterolemic and antiatherosclerotic effects of whole ground flaxseed supplementation along with a high cholesterol diet (HCD) in Wistar albino male rats.

## Materials and Methods

### Ethical approval

The approval of the Institutional Animal Ethical Committee was obtained before the commencement of the experiment.

### Procurement of experimental animals

Male Wistar albino rats weighing around 200 g were procured from Sri Venkateswara Agencies, Bengaluru. Rats were acclimatized to the experimental conditions for 1 week and were grouped and housed in standard polypropylene rat cages (three rats per cage) during the experiment. They were maintained at 25±1°C and a 12:12 h interval light/dark cycle and provided standard laboratory animal feed and *ad libitum* water throughout the experimental period of 90 days.

### Source of cholesterol and flaxseed

Cholesterol extra pure, AR grade with product code No: 97,900 was procured from the SRL fine chemicals, Indian Scientific, Tirupati, Andhra Pradesh. Dietary grade whole ground flaxseed was procured from the local market.

### Experimental design

A total of 48 healthy Wistar albino male rats were divided into four groups of 12 rats in each. Group I rats kept as control, Group II as positive control for induction of hypercholesterolemia and atherosclerosis by addition of 1% cholesterol and 15% saturated edible oil to the 1000 g of standard rat chew diet (HCD), Group III rats fed with whole grain flaxseed powder at 7.5 g/kg of rat/day in the standard rat chew diet and kept as flaxseed control, and Group VI rats fed with HCD along with flaxseed seed at 7.5 g/kg of rat/day and maintained for 90 days.

### Clinical observations

Health condition, behavior, and feed and water intake of all the rats were monitored throughout the experimental period. Body weights of the animals were recorded on the 45^th^ and 90^th^ days of experiment.

### Hematology

Blood samples were collected in 10% EDTA at each sacrifice from all the sacrificed rats and used for the estimation of total erythrocyte count (TEC), total leukocyte count (TLC), packed cell volume (PCV) by microhematocrit method [7], and hemoglobin (Hb) by Sahli’s method [8].

### Biochemical parameters

At each sacrifice, blood samples from all the groups were collected into the sterile test tubes. After blood clots, clear serum samples were separated without red blood cell and stored at 4°C. Estimation of TC, LDL-C, VLDL-C, HDL-C, and TG was carried out using commercially available biochemical kits (Auto Span diagnostics, Bengaluru).

### Tissue oxidative stress

Liver and heart tissue pieces were collected and stored at –20°C in the deep freezer until use. Tissue pieces of liver and heart were minced separately and homogenized in 0.05 M ice-cold phosphate buffer (pH 7.4) using a Virtis homogenizer to make 10% homogenate. For lipid peroxidation assay, 0.2 ml of the homogenate was used. The remaining part of homogenate was mixed with 10% trichloroacetic acid in the ratio of 1:1, centrifuged at 5000 g for 10 min at 4°C, and supernatant was used for the estimation of glutathione reductase (GR) [9]. The remaining part of the homogenate was centrifuged at 15,000 g for 60 min at 4°C, and the supernatant obtained was used for superoxide dismutase (SOD) [10], catalase [11], and glutathione peroxidase (GPx) [12] in liver and aorta of all rats in all groups.

### Histopathology

Small tissue pieces of aorta, heart, and liver were collected in neutral buffered formalin for routine histoprocessing by paraffin embedding technique and section was stained with hematoxylin and eosin [13].

### Statistical analysis

The results were analyzed statistically by performing one-way analysis of variance [14].

## Results

HCD-fed Group II rats clinically showed obesity with significant (p<0.05) increase in the body weight and rats were sluggish with poor hair coat, whereas flaxseed supplementation moderately reduced the obesity and body weight.

TEC, PCV, and Hb% of all groups (Groups I, II, III, and IV) were normal and non-significant (p<0.05) throughout the experimental period. TLC in Group II was nonsignificantly higher when compared to control Group I (Table-1).

**Table-1:** Mean values of body weight, serum biochemical, and hematological parameters of different experimental groups at the 45^th^ and 90^th^ days of experiment.

Parameters	Group I control		Group II positive control		Group III treatment control		Group IV treatment group	
			
	45^th^ day of study	90^th^ day of study	45^th^ day of study	90^th^ day of study	45^th^ day of study	90^th^ day of study	45^th^ day of study	90^th^ day of study
Body weight (g)	240±11.7	309.16±10.2	262.83±13.34	393.33±21.6^a^	242.33±9.47	325±13.4^b^	245±17.8	383.3±43.7^ac^
TEC (million/mm^3^)	5.78±0.9	6.30±0.2	6.6±0.45	6.83±1.3	6.41±0.30	6.7±0.5	7±0.60	7.46±1.02
TLC (x^mm3^ µl)	8.81±1.12	10.22±0.58	12.4±0.76	16.3±0.73	9.16±3.7	10.9±0.62	10.9±0.4	10.5±1.05
PCV (%)	34.6±5.7	37.83±1.3	39.6±2.6	32.5±2.1	38.5±1.8	37.83±1.7	40.5±3.4	41.3±1.2
Hb (g%)	11.55±1.92	12.61±0.4	13.2±0.8	10.8±0.7	12.83±0.6	12.61±0.5	13.5±1.15	13.7±0.4
TC (mg/dl)	46.03±7.8	50.67±7.04	113.58±11.75^a^	155.3±10.58^a^	48.75±2.75^b^	46.06±4.27^b^	99.0±6.9^abc^	83.8±4.1^abc^
TGs (mg/dl)	68.70±50.0	67.17±11.08	150.58±33.27^a^	178.2±39.84^a^	57.43±9.14^b^	67.83±34.93^b^	122.1±17.6^abc^	115.8±21.0^abc^
LDL-C (mg/dl)	17.44±4.7	20.13±2.78	64.88±5.86^a^	95.9±9.10^a^	16.31±2.85^b^	17.45±4.69^b^	30.4±2.9^abc^	28.9±2.4^abc^
Mean value of VLDL-C (mg/dl)	10.45±1.8	13.43±2.22	21.12±6.65^a^	31.0±8.78^a^	11.49±1.83^b^	11.65±5.32^b^	17.4±3.5^a^	14.2±4.2^b^
HDL-C (mg/dl)	28.16±2.8	27.10±5.15	17.58±5.35^a^	18.4±3.96^a^	29.95±2.93	28.96±3.02	22.2±2.4	21.7±2.6

Values are mean±SD, n=6. Values with different superscripts differ significantly (p<0.05) from the normal control or HCD or flaxseed control. TC=Total cholesterol, Hb=Hemoglobin, TGs=Triglycerides, VLDL-C=Very low-density lipoprotein cholesterol, TEC=Total erythrocyte count, PCV=Packed cell volume, TLC=Total leukocyte count, HDL-C=High-density lipoprotein cholesterol

TC, TG, LDL-C, and VLDL-C were significantly (p<0.05) increased, whereas HDL-C was significantly reduced in Group II rats fed with HCD. Tissue antioxidants such as catalase, SOD, GPx, GR, and glutathione S transferase (GST) were significantly (p<0.05) reduced, whereas lipid peroxidation products of thiobarbituric acid reactive substances (TBARS) level were nonsignificantly (p<0.05) increased in the heart and liver tissues of Group II. Flaxseeds supplementation reduced the elevated serum hyperlipidemic profile due to HCD by significant (p<0.05) reduction of TC, TG, LDL-C, and non-significant elevation of HDL-C. Flaxseeds nonsignificantly (p<0.05) increased all the cellular antioxidant enzymes measured in the experiment and reduced the level of TBARS both in the heart and liver tissues (Table-2).

**Table-2:** Effect of flaxseed (*Linum usitatissimum*) on tissue antioxidants at the 45^th^ and 90^th^ days of experiment.

Parameters	Group I control		Group II positive control		Group III treatment control		Group IV treatment group	
			
	45^th^ day of study	90^th^ day of study	45^th^ day of study	90^th^ day of study	45^th^ day of study	90^th^ day of study	45^th^ day of study	90^th^ day of study
TBARS (nmoL TBARS/g tissue) in the liver	1.38±0.42	1.36±0.27	1.8±0.32	2.02±0.23	1.34±0.19	1.03±0.24	1.48±0.32	1.52±0.26
TBARS (nmoL TBARS/g tissue) in the heart	1.98±0.26	1.84±0.32	3.4±0.23	4.1±0.17	1.78±0.19^b^	2.2±0.14^ab^	2.34±0.25^bc^	2.67±0.24^abc^
Catalase activity (nM of H_2_O_2_ decomposed/min/mg of protein) in the liver	0.25±0.020	0.28±0.020	0.15±0.03	0.14±0.02	0.24±0.03	0.25±0.02	0.16±0.05	0.17±0.06
Catalase activity (nM of H_2_O_2_ decomposed/min/mg of protein) in the heart	0.3±0.032	0.35±0.024	0.19±0.02	0.14±0.012	0.34±0.009	0.32±0.023	0.25±0.023^abc^	0.27±0.021^abc^
SOD activity (U/min/mg of protein) in the liver	18±1.1	16±1.8	14±1.02	12±2.2	17±0.9	18±1.8	15±0.9	14±2.3
SOD activity (U/min/mg of protein) in the heart	15±1.2	14±1.7	10±1.52	9±1.8	13±0.78^ab^	16±1.76^b^	12±0.8^ab^	13±1.8^b^
GPx activity (U/min/mg of protein) in the liver	28±1.1	26±1.6	22±0.9	20±1.2	27±1.3	28±0.8	25±1.6	24±1.2
GPx activity (U/min/mg of protein) in the heart	24±1.3	23±0.8	19±1.4	15±1.4	24±0.87^b^	23±1.56^b^	22±1.21^b^	19±1.32^abc^
GR (nmol of GSSG utilized/min/mg protein) in the liver	7.54±0.23	6.9±0.32	4.5±0.34	3.5±0.19	7.8±0.35^b^	7.1±0.43^b^	5.4±0.13^abc^	4.5±0.33^bc^
GR (nmol of GSSG utilized/min/mg protein) in the liver	9.77±0.34	8.8±0.24	5.5±0.28	4.8±0.21	9.5±0.33^b^	8.6±0.43^b^	6.3±0.16^abc^	6.9±0.18^abc^

Mean values with different superscripts differ significantly (p<0.05). TBARS=Thiobarbituric acid reactive substances. SOD=Superoxide dismutase, GPx=Glutathione peroxidase, GR=Glutathione reductase

Major gross and microscopic pathological changes were observed in the liver of Groups II and IV. Aorta from all the group rats found to be normal. Heart from few rats of Group II revealed slight enlargement and rounding.

### Liver

**Figure-1:**
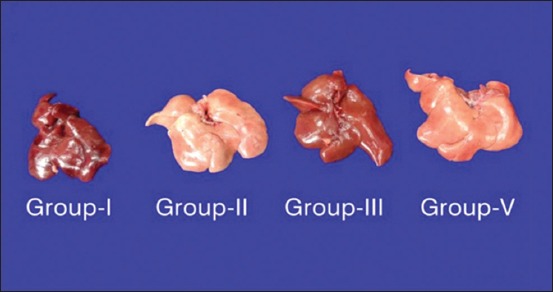
Liver: Note the reduced enlargement and paleness of flaxseed supplemented Group IV liver compared to high cholesterol diet Group II liver.

**Figure-2:**
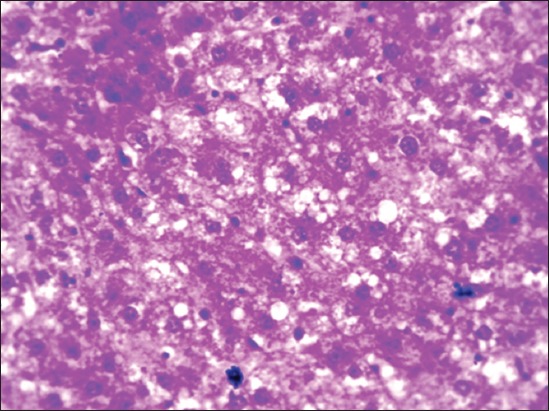
Section of Group II liver showing mild-to-moderate micro- and macro-vascular fat vacuoles in the hepatocytes (H and E, 400×).

**Figure-3:**
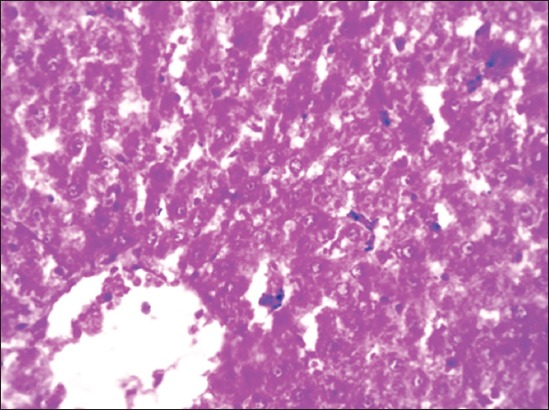
Section of Group IV liver showing few small fat vacuoles in the hepatocytes (H and E, 100×).

**Figure-4:**
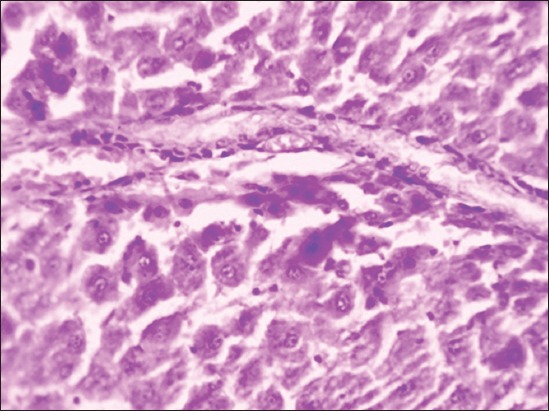
Group III liver showing proliferated endothelial cells forming vascular channels (H and E, 400×).

Group II rats’ liver was enlarged, soft, and pale in color indicating the fatty change and the degree of changes was higher in the 90^th^ day slaughtered rats compared to the 45^th^-day slaughter. Severity of enlargement and degree of paleness were very mild by the 45^th^ day and moderate by the 90^th^ day of flaxseed ameliorated Group IV**.** No specific gross changes were observed in control Groups I and III (Figure-1). HCD-fed Group II rats microscopically revealed varying sizes of fat vacuoles in various zones of the hepatocytes (Figure-2). These changes were very mild and less conspicuous in flaxseed ameliorated Group IV. Liver steatosis was absent in control Groups I and III throughout the study period (Figure-3). Proliferation of endothelial cells with formation new vascular channels were observed the liver of the 90^th^-day Groups III and IV (Figure-4).

### Aorta: HCD Group II

**Figure-5:**
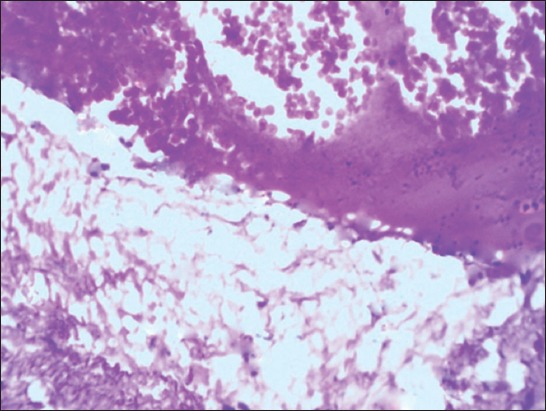
Aorta Group II section showing severe endothelial degeneration, foam cell accumulation with initiated atheromatous plaque (H and E, 400×).

**Figure-6:**
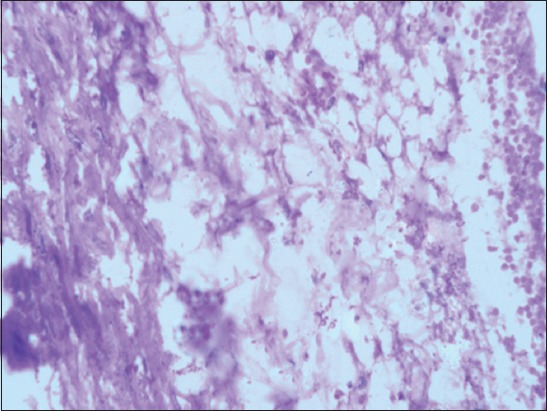
Aorta Group II section showing endothelial degeneration with subendothelial lipid-laden macrophages (foam cells) accumulation (H and E, 400×).

Aorta from HCD-fed Group II rats revealed initiation of atherosclerotic lesions with degeneration of endothelial cells, mild thickening of tunica intima, subintimal accumulation of scattered macrophages, and foam cells (Figures-5 and 6). Stromal cell proliferation with swollen nucleus and abundant cytoplasm was also observed in few cases by the end of experiment. Hemorrhage in between cardiac muscle fibers was also observed in few rats of atherogenic diet fed Group II.

### Group IV (HCD+flaxseed)

**Figure-7:**
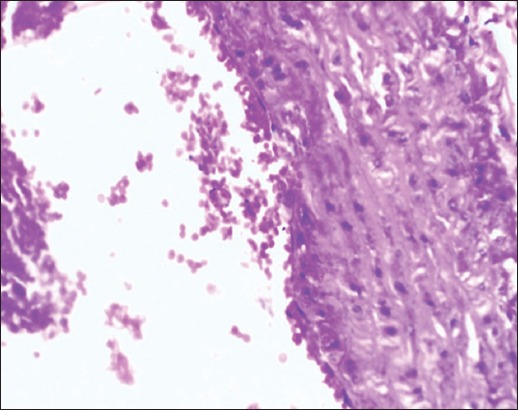
Aorta (Group IV) section showing mild endothelial degeneration with adhered erythrocytes (H and E, 100×).

**Figure-8:**
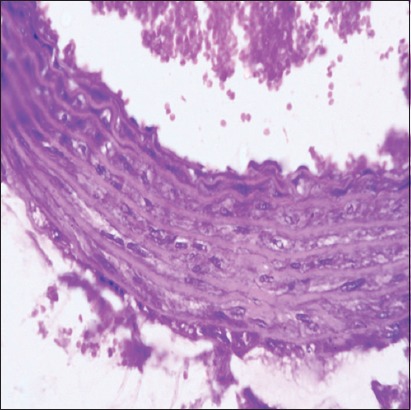
Group I aorta note normal endothelium with free erythrocytes in the lumen of aorta (H and E, 100×).

Mild-to-moderate endothelial degeneration, thickening of tunica intima with few foam cells was observed in the flaxseed ameliorated group by the 45^th^ day of experiment. Mild endothelial degeneration with adhesion of erythrocyte and few fat cells subintimally was seen on the 90^th^ day slaughtered rats (Figure-7). Mild endothelial cell proliferation with the formation of new vascular channels in between cardiac muscle fibers was observed throughout the study period of Groups III and IV, and it was very conspicuous by the 90^th^ day of experiment. Groups I and III aorta were normal without initiated atherosclerotic changes (Figure-8).

## Discussion

During the experimental period, no abnormal clinical symptoms were observed in control Groups I and III rats throughout the study period whereas obesity, sluggishness with poor hair coat was observed in the HCD-fed Group II rats [15]. Moderate obesity was observed in HCD supplemented with flaxseed Group IV rats.

Results in the present study revealed that rats on HCD (Group II) showed a non-significant (p<0.05) increase in the body weight in comparison with control Group I rats which received basal rat diet by the 45^th^ day and significant (p<0.05) increase by the end of experiment period [16,17]. It might be due to high calorie fat (1% cholesterol and 15% saturated oils) present in the HCD group compared to standard basal rat chew in control Group I. Non-significant (p<0.05) reduction in the body weight was observed in the flaxseed ameliorated Group IV throughout the experiment period when compared to HCD-fed Group II [18].

Clinical-pathological parameters such as TEC, PCV, and Hb of all groups (Groups I, II, III, and IV) were normal and non-significant (p<0.05) throughout the experimental period. TLC in HCD-fed Group II rats was nonsignificantly higher when compared to control Group I, and it might be due to increased level of LDL cholesterol which is responsible for increased viscosity of the blood and thereby resulted into high TLC [19,20]. The TLC levels were nonsignificantly (p<0.05) reduced in flaxseed ameliorated Group IV but not to the level of Group I. Reduced level of LDL cholesterol might have reduced the level of TLC in the flaxseed ameliorated Group IV.

Rats on HCD (Group II) showed a significant (p<0.05) increase in serum TC, TG, LDL-C, and VLDL-C and significant (p<0.05) decrease in HDL-C compared to control Group I rats that received standard basal diet [1,21], and it might be due to high-calorie fat diet compared to standard rat chew diet of control Group I. It indicated that the diet under trial had established a hyperlipidemia in this group of rats.

LDL is a lipoprotein that transports lipids from the liver to the peripheral (extrahepatic) and is often called “bad” cholesterol and constitutes a half to two-thirds of cholesterol [22] and high levels of LDLs are highly atherogenic lipoproteins. Oxidation of LDL in the walls of arteries may lead to an impaired endothelial relaxation in isolated arterial segments, thereby causing atherosclerosis [23]. HDLC is often called “good” because it is a lipoprotein that transports lipids from the periphery to all the liver. HDL particles enhance the net removal of cholesterol from a variety of cells such as smooth muscle cells, fibroblasts, and cholesterol-laden macrophages [24]. HDLs also prevent the oxidation of LDL by virtue of its antioxidant and anti-inflammatory properties [25]. The low levels of HDL in the blood will increase the risk of atherosclerosis and coronary heart disease [26].

Cotreatment with the flaxseed along with HCD (Group IV) significantly (p<0.05) reduced the TC, TG, LDL-C, and VLDL-C and nonsignificantly elevated the HDL-C when compared to HCD-fed Group II rats but not to the level of Group I by the end of experimental period [6,27,28]. Flaxseed Omega-3 fatty acids play a role in reducing the risk of CVDs by reducing hypertension, cholesterol, TGs, and free radicals. Flaxseed fibers also reduce the blood cholesterol levels by delaying and reducing their absorption from the intestines [5]. Flavonoids and phenolics of flax lignan complex are potent antioxidants and exhibit hypolipidemic and antiatherogenic effects, and it is synergistic with action of flaxseed SDG [6].

Results of the present study revealed significant (p<0.05) increase in TBARS levels in both liver and heart (includes aorta) of Group II rats compared to Group I, which indicates an increased amount of oxidative stress in the HCD-fed rats [29]. Hypercholesterolemia induces oxidative stress by causing a reduction in the tissue defense antioxidant enzymes, leading to acceleration of lipid peroxidation, cellular injury, atherosclerosis, and heart disease [30]. Antioxidant enzymes such as CAT, SOD, GPx, GR, and GST activity were reduced in Group II rats compared to control Group I rats fed on standard diet [15]. Addition of flaxseeds to the HCD in Group IV improved all the antioxidant enzymes CAT, SOD, GPx, GR, and GST and modestly reduced the levels of TBARS. It might be due to the antioxidant action of flaxseeds [6].

Gross changes in different organs are not conspicuous except enlargement and paleness of liver grossly and micro- to macro-vascular fatty vacuoles in hepatocytes microscopically in Group II rats, and it might be due to inclusion of 1% cholesterol, and 15% saturated fat to the rat diet [29,30]. The severity of enlargement and paleness and fatty vacuoles in the hepatocytes were very mild and less conspicuous in flaxseed ameliorated Group IV, and it might be due to antihyperlipidemic effects of flaxseeds [31,32].

Aorta from HCD-fed Group II revealed moderate initiation of atherosclerotic lesions with degeneration of endothelial cells, subintimal lipid-laden macrophages (foam cells), and slight thickening of the tunica intima with proliferation of few SMCs [1,17,29]. It might be due to oxidation of high level of serum LDL-C. Except mild endothelial degeneration, no other changes were observed in the aorta of rats supplemented with flaxseed (Group IV) by 90^th^ day of sacrifice. Flaxseeds are known to have varying antihyperlipidemic and antiatherogenic activity due to its lignans (SDG), ED, EL, alpha-linolenic acid, and omega-3 fatty acid components [33-35].

## Conclusion

HCD of the present study established the hyperlipidemia, thereby it initiated the atherosclerotic lesion in Group II rats but failed to form the complete atherosclerotic plaque, and it might be due to low level of cholesterol (1% cholesterol) and also due to short span of the study period. Flaxseeds supplementation reduced the hyperlipidemia to a certain extent caused by HCD; thereby, it might have prevented the initiated atherosclerotic lesions in the aorta, but not completely ameliorated the changes caused by hypercholesterolemia. Further, proliferation of endothelial cells was very conspicuous in the liver and heart of flaxseed control and supplemented group, and it might be due to its potent angiogenic effect and it needs to be explored further.

## Authors’ Contributions

This experiment was carried out by HSN under the guidance of CS KS, BS and TNVKVP assisted and guided in various parts of research, writing and corrections. All authors read and approved the final manuscript.
